# Supplementary material for: Phytochemical Profile and Antimicrobial Potential of Extracts Obtained from Thymus marschallianus Willd
Source: Molecules. 2019 Aug 26;24(17):3101. doi: 10.3390/molecules24173101 (PMC6749499; doi:10.3390/molecules24173101)
Supplement: Supplementary file 1 [file molecules-24-03101-s001.pdf]

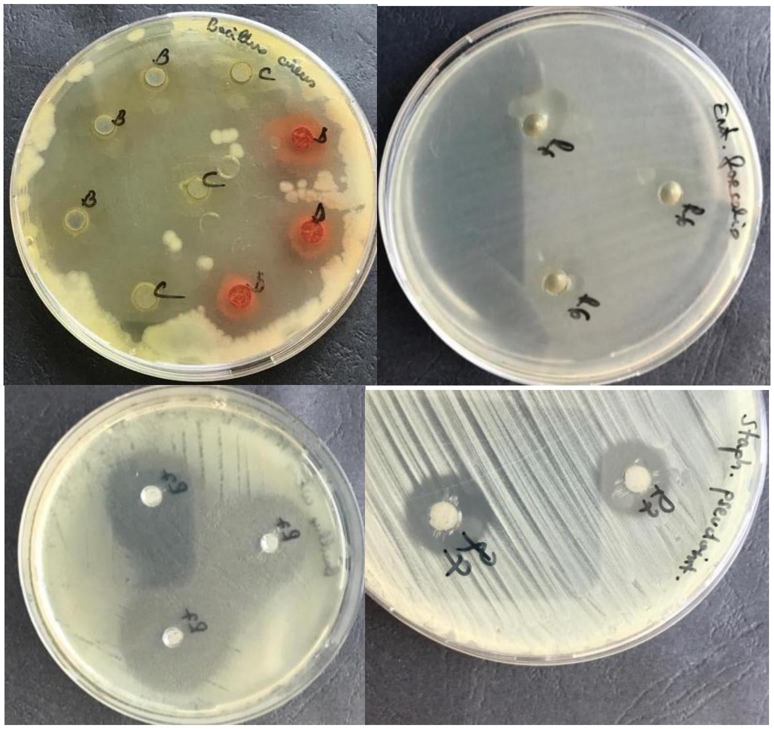

Figure 1. Antimicrobial preliminary screening using the well-diffusion method for TMs (B, R6) and TMc (C, R7)

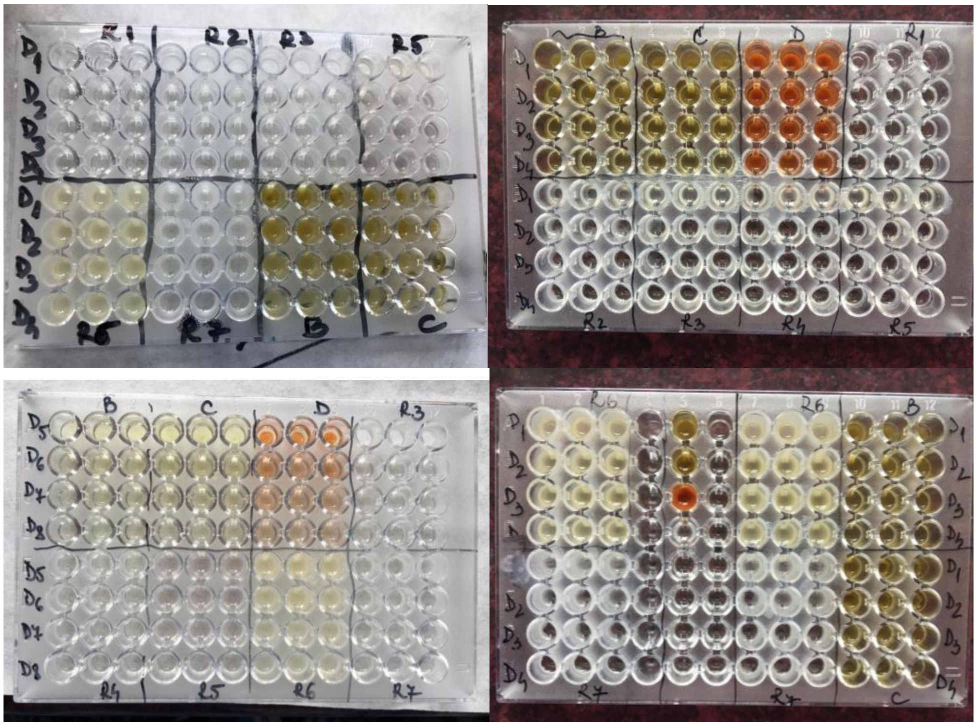

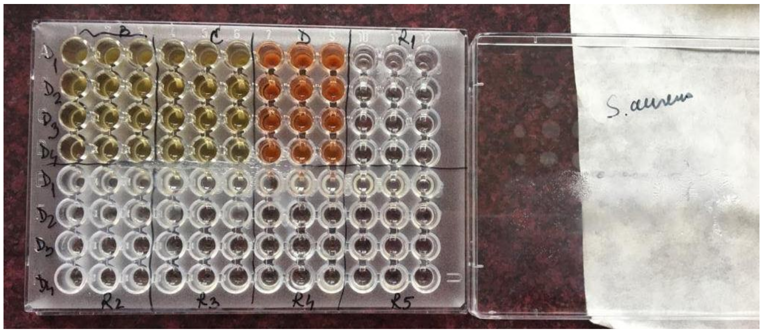

Figure 2. Minimum inhibitory concentrations (MICs) determined by a broth microdilution method for TMs (B, R6) and TMc (C, R7)

Notes: D1-D4 – tested dilutions from this plate

Each sample - TMs (B, R6) and TMc (C, R7) was placed in three wells (made in agar for the well-diffusion method and 96 plate wells for broth microdilution method) and Each evaluation was performed three times. The tested bacterial strains were the following: *Staphylococcus aureus* ATCC 25923, *Staphylococcus pseudintermedius* ATCC 49444, *Bacillus cereus* ATCC 14579, *Enterococcus faecalis* ATCC 29219, *Salmonella typhimurium* ATCC 14028 and *Salmonella enteritidis* ATCC 13076.
